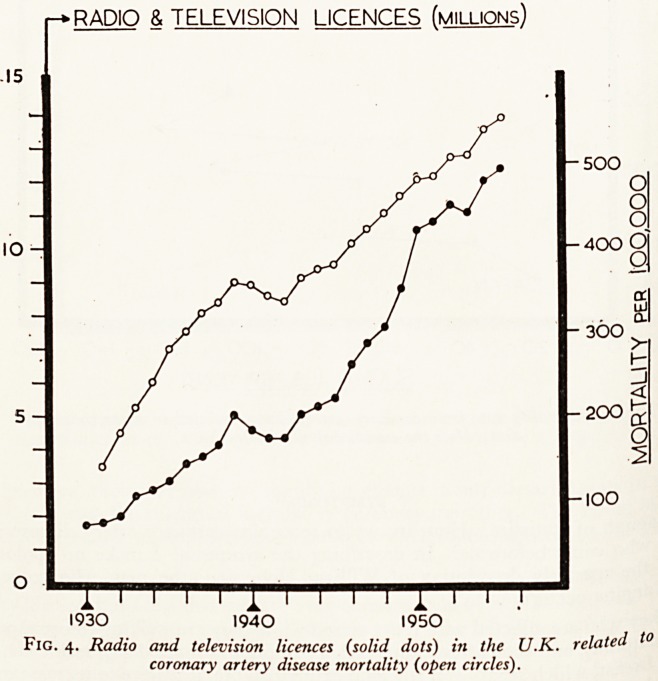# Cardiac Ischaemia
*Based on an address to the Bristol Medico-chirurgical Society.


**Published:** 1960-01

**Authors:** Gordon Mather

**Affiliations:** Consultant Physician, Bristol Clinical Area


### CARDIAC ISCHAEMIA*

BY

GORDON MATHER, M.D., M.R.C.P.
*Consultant Physician, Bristol Clinical Area*

It is with humility that one approaches a topic of which others have far greater experience but my reason for discussing cardiac ischaemia is that I have been so struck recently by its frequency. In my own practice in the last two years I have encountered no less than 318 cases and the annual toll from this disease in 1957 in England and Wales was over 76,000.

### HISTORY

It was Galen who named and described the coronary arteries, but William Harvey who appreciated their function. In 1649 he described a man "who did often complain of an oppression in his breast" and "at last opprest in a signal paroxism, he dyed"; the post-mortem examination showed aortic stenosis and a ruptured left ventricle presumably due to infarction. Much of the pioneer work on cardiac ischaemia has been done by Englishmen, such as William Heberden who in 1768 described "angina pectoris" and Caleb Parry of Bath, Edward Jenner of Berkeley and John Hunter of London who related the symptoms to narrowing of the coronary arteries. Jenner in describing the post mortem of a patient who died in Dursley in 1772 writes "My knife struck something so hard and gritty as to notch it. I well remember looking up at the ceiling, which was old and crumbling, conceiving that some plaster had fallen down. But on further scrutiny the real cause appeared: the coronaries were become bony canals". Parry belonged to a select medical society of five which included Jenner. In a meeting at Rodborough in 1788, he described "syncope anginosa" and related the symptoms to the morbid anatomy thus: "The rigidity of the coronary arteries may act proportionably to the extent of the ossification, as a mechanical impediment to the free motion of the heart, and though a quantity of blood may circulate through these arteries, sufficient to nourish the heart, as appears, in some instances from the size and firmness of that organ, yet there may probably be less than what is requisite for prompt and vigorous action".

### FREQUENCY

In 1908, only 929 deaths were certified as due to angina whereas in 1957 there were 76,324 deaths attributed to coronary heart disease. This figure would be even higher if we included the 50,000 people on whose death certificates "myocarditis" or "myocardial degeneration" was written as the cause of death. Some of this enormous increase must be due to more accurate clinical diagnosis with the increased use of the electro-cardiogram following the pioneer work of Eindhoven, Pardee (1920), and Parkinson and Bedford (1928). Other causes are an ageing population and changing of fashion in diagnosis. But there is overwhelming evidence of a very real increase in mortality from coronary artery disease. The death rate from it has doubled since 1947, and nearly 15 per cent of all deaths are now due to it. Coronary artery disease kills nearly as many people each year as cancer in all its forms and sites. Yet I wonder how many mites are spent on research on this disease compared with the gold coffers of the cancer research fund.

* Based on an address to the Bristol Medico-chirurgical Society.

The ratio of mortality in men to that in women varies from 1·4 in Japan to 4·4 in Finland: in the U.K. it is 1·68. Occupation seems to affect mortality: the standardized mortality rate in U.K. is very high among physicians and surgeons (368), barristers and solicitors (227), employers and managers (190) compared with coalminers (40) and agricultural labourers (32). This can be interpreted in several ways: it may reflect the selection for the professions of the most progressive and restless members of the community, or the burden of responsibility which they carry, or a lack of physical exercise or an increased consumption of tobacco and animal fat! Nevertheless, it is a sobering thought that the cause of death of the professional and managerial sections of our community—readers of *The Times*—will in more than a third be coronary artery disease. However, the notion that agricultural workers are less prone to coronary disease may be wrong—Kennedy (1957) found that 24 per cent of his admissions with this disease in Dumfries were farm workers, a proportion corresponding to the population served. It would seem that they have a better prognosis.

Brown and others in Birmingham (1957) showed that the frequency of symptoms from coronary artery disease is higher in sedentary than manual workers. There were also more myocardial infarcts in the professional compared with the unskilled occupations though the prevalence of angina was higher in the manual worker. It is this curious dissociation of cardiac infarcts from angina that has always intrigued me; ischaemic heart disease is not a simple function of coronary artery disease. Jamaican negroes for instance have coronary atheroma as frequently as the white population of the Southern U.S.A., but only rarely suffer from ischaemic heart disease, (Robertson, 1959). Further evidence on this point comes from Morris and Crawford (1958) when reporting a national necropsy survey of the disease. They found no variation among different occupations in the frequency of coronary atheroma or stenosis; ischaemic myocardial fibrosis and coronary occlusions were however, more common in those with light occupations compared with heavy ones, and appeared at an earlier age. There is however one criticism of this work, that the occupations referred to are always the last stated ones—and a man with coronary disease may well change to a lighter job late in life. Nevertheless, should we not perhaps think of ischaemic heart disease as a *deficiency disease*—a deficiency of exercise?

Over-indulgence in alcohol does not seem to be an important factor in coronary artery disease. Barmen and French wine workers show a high mortality rate from cerebro-vascular diseases and chronic nephritis, but only a slight increase from coronary artery disease. Cigarette smoking does seem to be associated with a higher mortality from this disease—about double for those who smoke 20 or more daily (Hammond and Horn). But I do not think that this relationship need be one of cause and effect; both habit and disease may be an expression of a man's emotional make-up.

Hereditary factors are undoubtedly important in this disease—the body build, the coronary arteries themselves and the degree to which they anastomose with each other, the metabolism and the nervous balance of the individual.

### DIETARY FAT

There are two ways in which an excess of dietary fat may influence the disease, either by accelerating the development of atheroma, or by provoking intravascular thrombosis—though the two may be related if Duguid's work (1946) is accepted . . . Firstly let us consider the development of *atheroma*.

(1) In experimental rabbits, cholesterol deposition on the arterial wall can be directly related to the height and duration of hypercholesterolaemia artificially induced by a diet high in cholesterol. From this evidence it has been argued that in man, in whom atherosclerosis develops spontaneously, control of the serum cholesterol is both desirable, and beneficial in coronary artery disease. I think this deduction is quite unwarranted.

(2) Patients with established coronary disease are said to have a higher mean level of serum cholesterol than normal subjects (Oliver and Boyd, 1956) and to have a greater and longer lipaemia after eating butter (Barritt, 1956) but these may be the effects, or related abnormalities, rather than the cause of the disease.

(3) Patients who have hypercholesterolaemia, such as diabetics and those with rare familial diseases such as Xanthoma tuberosum, succumb to coronary artery disease more frequently than normal people. This may be so, but it is quite a different matter to infer from this that reduction of the blood cholesterol is therefore desirable or beneficial.

(4) It has been shown that the serum cholesterol can be reduced by a change from animal to vegetable fats (Kinsell 1952, Bronte Stewart 1955). At first this effect was thought to be due to essential fatty acids, then to sitosterol, but now the accepted view is that it is due to the unsaturated fatty acid content of the vegetable fats. These fats are only effective when substituted for saturated fats, not when merely used as supplements. In other words it is no use having potatoes fried in sesame oil and corn oil salad dressing in a meal which starts with pâté de foie gras, progresses to roast pork and ends with strawberries and cream and a slice of Stilton! However, it has yet to be shown that reduction of serum cholesterol has any effect on the prognosis of coronary artery disease.

Secondly, does dietary fat have any effect on *blood coagulation*?

(1) In Aberdeen, Fullerton (1953) showed that there is a shortening of the silicone clotting time after a fatty meal, which is greater in convalescent ward patients than in sedentary students. Mersky and Nossel (1957) on the contrary found no effect on blood coagulation of diets differing in their fat content.

(2) In London some workers (McDonald and Edgill (1957)) showed a difference in various clotting factors between patients with ischaemic heart disease and controls. There was however a considerable overlap and no conclusive evidence was obtained that the hypercoagulability was not the effect rather than the cause of the disease. By contrast O'Brien (1958) in Portsmouth found no difference in the blood coagulation of patients with coronary disease compared with controls after a fatty meal. As he says: "There is no support for the hypothesis that postprandial hypercoagulability is responsible for the alleged connection between the fat content of the diet and coronary thrombosis.

(3) Other work has been done by Greig (1956) on fibrinolysins showing that they are increased after exercise and decreased after a fatty meal. But this work has not been confirmed by other workers.

(4) It can thus be seen that there is much conflicting evidence concerning the effects of fat on blood coagulation. It is important to remember the work of Master et al (1939) who showed that the time of onset of coronary thrombosis was evenly distributed throughout the 24 hours, and thus unrelated to the major meals. Also no thrombosis is found in more than one third of all those dying from infarcts.

Dietary habit studies do not help us to decide about the role of fat. Evidence based on the incidence of coronary artery disease in primitive tribes cannot really be accepted as their life expectancy is so much less than in so-called civilized communities, standards of medical diagnosis are far below ours, and racial factors may be important. Indeed where statistics have been collected carefully as for example by Malhotra and Pathania (1958) from the Punjab, they show that coronary artery disease in India is not a rarity—it formed 28·4 per cent of their cardiac patients. This is even more remarkable when we appreciate that most of these patients were on a low fat diet, many were vegetarians and non-smokers! The figures of Professor Keys (1953) of

Minnesota are often quoted to show that nations with a high consumption of fat (such as the U.S.A.) have a high incidence of coronary artery disease compared with Japan where fat consumption is low. But as Yudkin (1957) showed, although the U.S.A., U.K. and Norway had similar fat intakes, their mortality rates from coronary artery disease varied enormously. (See Fig. 1.) A comparison with Fig. 2 shows that there is almost as close a relationship to sugar consumption.

FIG. 1. *Mortality rate from coronary artery disease related to fat consumption. Each dot represents a country: only a few are named for the sake of simplicity.*

Furthermore, there has been no significant change in our dietary habits in the last 20 years to match the relentless increase in coronary mortality. The rising mortality can in fact be related more closely to the number of television licences! (See Figs. 3 and 4).

I am unable to accept a theory which supposes any single or major dietary cause of coronary disease, but I feel that relative over-consumption of food and reduced physical exercise may be contributory causes of the disease. The fall in mortality in Norway during the German occupation may well have been due to reduction of total calorie intake and increased exercise. Until we have further evidence on this subject of diet in coronary disease, I do not feel we are justified in altering our habits. Nor can we be sure that these unsaturated vegetable fats which some would advocate do no harm; in rats they cause gastric ulcers and even carcinoma. And the mortality rate from gastric carcinoms aged 55–64 in Sweden is two to three times that in the U.S.A.—perhaps due to greater consumption of unsaturated fish oils? In short let us be cautious that we do not jump from our fatty frying pans into the fire of cancerous complications of corn oil.

FIG. 2. *Mortality rate from coronary artery disease related to sugar consumption. Note the essential similarity to Fig. 1.*

### SYMPTOMS

But enough of statistics. How are we to recognize coronary artery disease in the patients who come before us? In describing the symptoms I make no apology for quoting the masterly description of William Heberden, the man who coined the phrase "angina pectoris".

"They who are afflicted with it are seized while they are walking (more especially if it be uphill), and soon after eating with a painful and most disagreeable sensation in the breast, which seems as if it would extinguish life, if it were to increase or continue, but the moment they stand still, all this uneasiness vanishes.

FIG. 3. *Animal fat consumption (solid dots) in the U.K. during the last forty years related to coronary artery disease mortality (open circles).*

FIG. 4. *Radio and television licences (solid dots) in the U.K. related to coronary artery disease mortality (open circles).*

(*Figs. 1–4 adapted from Yudkin,* Lancet 1957, *by kind permission.*)

"In all other respects, the patients are, at the beginning of this disorder, perfectly well, and in particular have no shortness of breath, from which it is totally different. The pain is sometimes situated at the upper part, sometimes in the middle, sometimes at the bottom of the os sterni, and often more inclined to the left than to the right side. It likewise very frequently extends from the breast to the middle of the left arm. The pulse is, at least sometimes, not disturbed by this pain, as I have had opportunities of observing by feeling the pulse during the paroxysm. Males are most liable to this disease, especially such as have passed their fiftieth year.

"After it has continued a year or more, it will not cease so instantaneously upon standing still: and it will come not only when the persons are walking, but when they are lying down, especially if they lie on the left side, and oblige them to rise up out of their beds. In some inveterate cases it has been brought on by the motions of a horse, or a carriage, or even by swallowing, coughing, going to stool, or speaking; or any disturbance of mind."

Could anyone today really improve on this description? I would only like to emphasize the importance of emotional excitement in producing pain, and one experience which seems to be very provocative is watching a boxing match on television. One patient of mine gets angina when his wife drives the car! I wish also to say how many patients I have seen in recent years in whom the pain of an infarct has been in the left, or occasionally the right mammary line and not substernal. Pain below the costal margin has only been rarely encountered, also pain in the arm alone unless accompanied by chest pain. And remember that in some patients Trinitrin tablets give no relief, presumably because the peripheral vessels relax more than the coronaries.

### DIAGNOSIS OF CARDIAC PAIN

The diagnosis of angina rests almost entirely on an accurate history. Physical examination and radiography seldom help, and the E.C.G. helps in only a small proportion. The family doctor who knows the previous personality and family background can best assess whether the symptoms stem from anxiety about the heart or from coronary artery disease. Confusion with *Hiatus Hernia* may occur but symptoms from the latter usually have an association with eating or posture, and it must be remembered that the demonstration of a hernia does not necessarily mean that it is the cause of the symptoms. This remark also applies to *Cervical Spondylosis:* pain from this common condition may pass round to the front of the chest as well as down the arm, but there is usually a history of neck pain and of a wrench of the arm or neck at the onset. Symptoms from a diseased *Gall Bladder* may exist at the same time as those from cardiac ischaemia, and there is interesting experimental evidence that distension of a hepatic duct may cause a reflex fall in coronary blood flow. It can sometimes be a nice clinical problem to decide whether a diseased biliary system is contributing to cardiac symptoms. Gall Bladder pain may be midline, but is seldom felt in the chest, and is not related to effort.

When a diagnosis of cardiac pain has been made, this is not tantamount to saying that coronary atheroma is the only disease process present. *Anaemia* must always be considered and haemoglobin estimation done if there is any doubt. Many patients obtain complete relief when the anaemia is corrected. *Aortic Stenosis* or incompetance may be the cause of cardiac pain, but relief here is most difficult. *Hyperthyroidism* by increasing the work of the heart may cause pain. A patient of mine with acromegaly and hyperthyroidism required 20 trinitrin tablets daily in order to control angina. After treatment with neomercazole and later thyroidectomy she needed none. *Paroxysmal Tachycardias* may cause cardiac ischaemia, and their control by digitalis, quinidine or procaine amide usually brings relief from pain. *Syphilitic Aortitis* is now rare but it may cause cardiac pain by narrowing of the coronary ostia. Attempts to relieve the pain by anti-syphilitic treatment are usually unrewarding.

Occasionally cardiac pain occurs during *Rheumatic Fever* probably because of coronary arteritis and a youth whom I saw recently had this pain when cycling as the first evidence of carditis.

### DIAGNOSIS OF CARDIAC INFARCTION

Under what circumstances can we make a diagnosis of *Cardiac Infarction*, which in about two-thirds of patients is preceded by a coronary thrombosis?

The diagnosis can usually be suspected on clinical grounds: the persistence of pain, the presence of shock or syncope, the appearance of arrhythmia or heart failure. A fall in blood pressure does not always occur—sometimes there is a transient rise perhaps due to stimulation of medullary vaso-motor centres.

Tachycardia is not invariable—sometimes there is bradycardia due to reflex vagal stimuli or to a degree of heart block. The pain usually makes the patient want to keep still, but I have recently seen two patients who gained some relief by walking about the bedroom.

The electrocardiogram helps in the diagnosis in about 95 per cent of patients in their first attack. Sometimes if a record is taken too soon after the onset, typical changes will not have occurred, but they usually do so within 24 hours. Quite a proportion of infarctions show only on the precordial leads, which may explain why some were missed in the days when only limb leads were taken.

Other aids to the diagnosis are fever which usually occurs within three days, rise of E.S.R. and white cell count, and a rise in the serum glutamic oxalacetic transaminase. This enzyme is present in high concentration in cardiac and skeletal muscle, liver, brain and kidney; death of cells in these organs causes a liberation of enzyme into the blood. The normal level of this transaminase is up to 40 units, and the maximum rise after an infarct occurs between 12 and 48 hours after its onset. The estimation is of considerable value in deciding whether a recent infarction has occurred in a patient who has suffered previous episodes and in whom the E.C.G. is distorted by these or by bundle branch block. It may also help to determine whether an extension of infarction has occurred.

### TREATMENT

In discussing the treatment of angina may I remind you that a resident medical officer in Edinburgh, Lauder Brunton, first introduced amyl nitrite to the world in 1867. Eight years later in London, William Murrell (1875) observed the properties of nitroglycerine by using it on himself in out-patients, and he became quite alarmed by its effect!

### *Treatment of the Attack of Angina*

There has been no better trial of drugs for treatment of angina than that of Evans and Hoyle in 1934. They showed without any doubt that glyceryl trinitrate sublingually was the most effective drug for the relief of an attack (86 per cent of patients obtain great relief) and for the prophylaxis of an expected attack (84·5 per cent). No other drug has yet been shown to be more effective. Trinitrin is cheap, (5 shillings per 1000 tablets), reliable, and may be taken as frequently as half-hourly without any complications. It is important to emphasize to our patients that the tablets should be crunched and allowed to dissolve in the mouth. Recently a very stable product has come on to the market so that our supplies are now white rather than chocolate coloured.

*Prevention of Angina*

Evans and Hoyle showed in their extensive studies that most drugs were ineffective in the prevention of angina pectoris and they pointed the most valuable moral that 40 per cent of patients are improved by a placebo. They tried erythrytol tetranitrate and showed this to be of no value, and I have no reason to differ from their opinion, with regard to the more recent products, Mycardol and Peritrate which are pentaerythrytol tetranitrate. Nor have I found that the use of Praenitrona prevents attacks. There is no evidence that Vitamin E, sex hormones or iodides exert any beneficial effect on the course of the disease. Iproniazid has been found by Towers and Wood (1958) to give relief of pain but has serious toxic effects on the liver and other organs and hence can only be recommended in very severe angina.

In my opinion, the frequency of anginal attacks is best lowered by general measures such as reduction in weight, relief of anxiety where possible (sometimes with the aid of small doses of barbiturates), the avoidance of large meals and exercise after them, resisting the temptation to sweep snow or pull sledges, and a general slowing of the tempo of life, such as dropping a few committees and directorships. Any anaemia should of course be corrected. Trinitrin tablets when sucked before an exercise such as climbing stairs will prevent anginal pain. Nocturnal attacks can sometimes be prevented by patients taking a light supper, sucking Trinitrin when retiring to bed and taking a grain of phenobarbitone, or papaverine. Some advocate depressing the metabolism by anti-thyroid drugs, but the resultant mental and physical slowing may be as unpleasant as the angina.

*Treatment of Cardiac Infarction*

In treating this condition we are dealing with a patch of dead muscle which may by its presence cause:

(1) an electrically unstable heart leading to ventricular tachycardia or fibrillation which may be fatal,

(2) heart failure,

(3) cardiac aneurysm or rupture.

The aims of treatment therefore are:

(1) healing of the infarct and formation of a firm scar,

(2) the development of collateral blood supply to the surrounding muscle, and possible recanalization of a coronary thrombus,

(3) relief of pain,

(4) correction of hypotension, heart failure and arrhythmias

(5) prevention of thrombo-embolic complications.

Physical and mental rest is essential, and I consider that six weeks in bed, followed by a month's convalescence is advisable with perhaps some remission of sentence for the elderly and second offenders. I think a patient should not be moved in the shocked stage, unless he is in an unsuitable place, such as a train, theatre or the open air, as this may increase the shock and accelerate death.

Usually these people are best nursed in bed, but if they become very breathless, sitting up in a comfortable fireside chair may give most relief. A bedside commode or a sani-chair should be provided and a suitable bed-table to enable the patient to eat, read and write comfortably. A suitcase, hassock or other support for the feet in bed is an aid to comfort and leg movements. An important part of the management is *explaining* the position to the patient so that he is resigned to the period of rest advised and does not "champ against the bit". Provision of adequate interests after the first few days, such as books, radio or a hobby such as sketching or needlework are common sense and need not be glorified by the name "occupational therapy".

*Relief of pain* is usually effected by morphia, but I would like to commend the use of Pethidine, 150 mgm. and Largactil 50 mgm., certainly in those who vomit after morphine. Severe pain may last for several days, and these analgesics should not be spared. Thereafter occasional anginal pains may be relieved by Trinitrin. If there is much anxiety, phenobarbitone or amytal may help, and a nightcap of whisky does not go amiss.

Severe hypotension following a cardiac infarct carries a mortality rate of about 80 per cent. Improved prognosis results from the use of pressor agents. The most useful is mephentermine sulphate ("mephine") given intravenously or intramuscularly in doses of 15 to 30 mgms. L-noradrenaline is seldom needed and can only be given in hospital as careful supervision is necessary. But I do not consider the urgent removal of a severely shocked patient in a cold ambulance is beneficial, and wherever possible initial shock should be corrected at home.

*Oxygen* is widely used for the relief of the cyanosis and shock following an infarction, but there are theoretical objections to its use. In dogs it has been shown that by increasing the difference in oxygen tension between the infarcted and healthy muscle, the heart is made electrically unstable, and the risk of fatal ventricular fibrillation is increased. In consequence, I do not use oxygen unless there is dyspnoea and the patient is obtaining relief from it. One sees only too frequently oxygen being pressed on to a patient with no effect other than to make him struggle.

*Digitalis* is best avoided in the early days after an infarct unless a fast auricular fibrillation develops with incipient heart failure, when the risks of withholding the drug are greater than the risks of precipitating ventricular tachycardia. If multiple extrasystoles should occur during the course of an infarction, I do not hesitate to use *quinidine* as this may well prevent the development of a fatal ventricular tachycardia or fibrillation.

Should heart failure develop, then we should exhibit diuretics.

### *Anticoagulants*

There is still considerable discussion about the value of these drugs in coronary artery disease. Some still regard them as rat poison, others as the elixir of life. Gilchrist and Tulloch (1956) have shown that the mortality in a large series treated in hospital was halved by the use of anticoagulants. They do not claim that they can prevent heart failure, arrhythmias, or all recurrences. The main benefit is in the prevention of complicating venous thrombosis in the legs which is such a hazard on account of the later risk of pulmonary embolism.

The drawbacks are that:

(1) the drugs need to be started in hospital,
(2) they require frequent laboratory tests for control,
(3) they increase the risk of haemorrhage from silent peptic ulcers, or from the heart causing a haemopericardium.

But there is no doubt in my mind that the benefits of the drugs to a patient in hospital outweigh these disadvantages. All my patients in hospital who have a myocardial infarct receive anticoagulants for the duration of their stay, unless they are very old, or give a history of an active gastric or duodenal ulcer or colitis. The most difficult problem is whether to admit a patient for treatment with "dindevan" or treat him at home. I think the decision can only be made by considering all the factors—age, social condition, mental state, practicability of home nursing, severity of infarct and so on. I do think that patients under the age of 55 should be admitted for anticoagulants. There is increasing evidence that the frequency of recurrence can be reduced by long term anticoagulants. Manchester (1957) treated 204 patients who had suffered infarcts with anticoagulants for from 1 to 10 years and 200 controls with ascorbic acid for the same period. He found the incidence of subsequent infarcts three times greater and the mortality eight times greater in the control group compared with the treated group. Bleeding complications occurred in only 2·9 per cent. We await with interest the present Medical Research Council trial of this treatment*.

The difficulties of management are great, so I usually compromise and try to keep only those who have suffered more than two infarctions on these drugs indefinitely.

### SURGERY OF CORONARY ARTERY DISEASE

In general, a patient with severe coronary artery disease is a bad surgical risk: and major operative procedures are best avoided. Obviously the risk will often have to be taken, for instance when dealing with intestinal or prostatic obstruction.

What I am mainly concerned with is whether surgery has any part to play in the relief of angina. *Thyroidectomy* may occasionally be indicated if a toxic goitre is responsible for cardiac pain, but is not advisable for the relief of angina. *Sympathectomy* of the upper four thoracic ganglia has been practised where intractable pain is a problem. Obviously removal of the warning signal of pain may be dangerous and may tempt the patient to over exert himself but these patients are usually left with a feeling of substernal oppression as warning without frank pain. Benefit from sympathectomy may also come from the blocking of noxious reflexes which result in ventricular fibrillation. Lindgren (1950) gave relief of pain to three-quarters of 105 patients after sympathectomy, but did not claim any prolongation of life thereby.

Another group of operations aims at increasing the amount of blood reaching the heart by means of omental, jejunal, skin or lung grafts or arterialisation of the coronary sinus. O'Shaughnessy (1935) in this country and Beck (1935) in the U.S.A. were pioneers in this field but in general the results were disappointing and the mortality high. Vineberg (1946) in Canada has practised *internal mammary artery implantation* extensively, and in 1955 claimed that half of 30 survivors from the operation were completely or greatly relieved of their symptoms. Others have not so far been able to achieve such good results.

There is no limit to the ingenuity of our surgeons, and recently (1953) Gordon Murray has even attempted excision and grafting of atheromatous sections of the coronary arteries and excision of old infarcts (1947). Another attempted to repair a ruptured interventricular septum. Glover (1957) has practised simple *ligation of the internal mammary arteries* beyond where they give off their pericardial branches with the aim of increasing the blood flow. Although benefit is claimed in 68 per cent, there is considerable doubt of its value apart from it being a powerful mode of suggestion. Mention is made above of Evans and Hoyle's work showing that 40 per cent of patients obtain relief from a placebo.

The most promising field of surgery lies in stimulating the *inter-coronary anastamoses*. As long ago as 1669, a Cornishman, Richard Lower, described these anastomotic vessels. Zoll, Wessler and others in 1951 found these anastomoses in 9 per cent of normal hearts, 63 per cent of hearts with marked coronary narrowing and in 100 per cent where there were old occlusions. Zoll concludes that anoxia is the stimulus for the development of collateral vessels. The post-mortem arteriograms of Fulton (1956) in Edinburgh illustrate how extensive a network of collaterals may be developed in patients with coronary artery disease.

In experimental dogs there is electrical stability when either the heart is uniformly well oxygenated or uniformly cyanosed. But ligation of one coronary artery in a healthy heart or cannulation of one artery with oxygenated blood in a cyanosed heart produced the "current of injury", perhaps more correctly called the "current of oxygen differentials". It is under these circumstances that ventricular fibrillation may arise and the dog or patient "electrocute himself". *Beck's operation* is based on extensive experiments on dogs and aims at the development of sufficient collateral blood supply to prevent these dangerous differentials in oxygen tension. Beck (1935, 1958) has shown

* Now published in the *British Medical Journal*, March 28, 1959.

that most deaths in coronary artery disease are not due to massive infarcts or heart failure, but are from serious disturbances of rhythm, particularly ventricular fibrillation. This is supported by coroners' pathologists who frequently find in cases of sudden death that there is only early coronary artery disease and no obvious infarction. Dr. N. J. Brown tells me that in 100 consecutive post mortems for sudden deaths which were attributable to coronary artery disease, large infarcts were found in only 55, and recent thrombosis in only 58. Beck's operation consists of partial ligation of the coronary sinus, abrasion of the pericardium and insertion of asbestos powder. It should be emphasized that it has no effect on the underlying atheroma. In the last 347 cases operated on by Beck, there was a 6 per cent operative mortality, and of the survivors 32 per cent had great relief and 62 per cent moderate relief of cardiac pain. This 94 per cent were able to resume work. Mr. Michael Wilson who was trained by Beck, has done a few of these operations in Bristol. Obviously at first we have only offered the operation to those most seriously afflicted, and hence with the worst prognosis, but there have been several who have derived great benefit. One of these was a 61-year-old housewife who had such severe angina that she was confined to her room. A Beck operation was performed two years ago at Southmead Hospital and she is now able to enjoy a normal social life, going for walks of up to a mile.

It is my opinion that a patient should be considered for surgical treatment if he has incapacitating angina, providing he has no cardiac enlargement, hypertension, heart failure or evidence of recent infarction.

### PROPHYLAXIS

As individual doctors, and indeed potential patients ourselves what should we advise?

(1) Take regular physical exercise, but resist the temptation to do the work of a bulldozer after months of hibernation.

(2) Watch your vital statistics—don't dig your grave with your teeth.

(3) Don't give the Chancellor of the Exchequer a surplus in excise tax by your consumption of tobacco.

(4) What may be the most important factor—choose your parents carefully!

### SELECTED REFERENCES

Barritt, D. W. (1956). *Brit. Med. J.*, **2,** 640.
Beck, C. S., Leighninger D. S., Brofman, B. L., and Bond, J. F. (1958). *J. Amer. Med. Ass.*, **168,** 2110.
Beck, C. S., Tichy, V. L., and Moritz, A. R. (1935). *Proc. Soc. Exper. Biol. & Med.*, **32,** 759.
Bronte Stewart, B., Keys, A., and Brock, J. F. (1955). *Lancet*, **2,** 1103.
Brown R. G., Davidson, L. A. G., McKeown, T., and Whitfield, A. G. W. (1957). *Lancet*, **2,** 1073.
Brunton, T. L. (1867). *Lancet*, **2,** 97.
Duguid, J. B. (1946). *J. Path. Bact.*, **58,** 207.
Eindhoven, W. (1903). *Pflugers' Arch. F.d. Physiol.*, **99,** 472.
Evans, W. and Hoyle, C. (1934). *Quart. J. Med.*, **27,** 105.
Fullerton, H. W., Davie, W. J. A., Anastasopoulos, G. (1953). *Brit. Med. J.*, **2,** 250.
Fulton, W. F. M. (1956). *Brit. Heart J.*, **18,** 341.
Gilchrist, A. R. and Tulloch, J. A. (1956). *Scot. Med. J.*, **1,** 1.
Glover, R. P. (1957). *J. Arkansas Med. Soc.*, **54,** 223.
Greig, H. B. W. (1956). *Lancet*, **2,** 16.
Hammond, E. C. and Horn, D. (1954). *J. Amer. Med. Ass.*, **115,** 1327.
Harvey, W. (1649). *Exercitatio Altera.* Frankfurt.
Heberden, W. (1768). Lecture at R.C.P., London, 21st July.
Jenner, E. (1827). *Life of, J. Baron*, **1,** 39.
Keys, A. (1953). *J. Mt. Sinai Hosp.*, **20,** 118.
Kinsell, L. W., Partridge, J. W., Boling, L. A., Balch, H. E., and Cochrane, G. C. (1952). *J. Clin. Endocrin. & Metab.*, **12,** 909.

Lindgren, I. (1950). *Acta Med. Scand.*, **138**, (suppl. 243), 1.

Lower, R. (1669). *Tractatus de Corde*, London.

Malhotra, R. P. and Pathania, N. S. (1958). *Brit. Med. J.*, **2**, 528.

Manchester, B. (1957). *Ann. Intern. Med.*, **47**, 1202.

Master, A. M., Dark, S., and Jaffe, H. L. (1939). *Amer. Heart. J.*, **18**, 434.

McDonald, L. and Edgill, M. (1957). *Lancet*, **2**, 457.

McDonald, G. A. and Fullerton, H. W. (1958). *Lancet*, **2**, 600.

Medical Research Council (1959). *Brit. Med. J.*, **1**, 803.

Merskey, C. and Nossel, H. L. (1957). *Lancet*, **1**, 806.

Morris, J. N. and Crawford, M. D. (1958). *Brit. Med. J.*, **2**, 1485.

Murrell, W. (1875). *Lancet*, **1**, 80, 113, 151, 225.

Murray, G. (1953), (1947). *Ann. Surg.*, **126**, 523.

O'Brien, J. R. (1957). *Lancet*, **1**, 1213.

Oliver, M. F. and Boyd, G. S. (1956). *Lancet*, **2**, 1273.

O'Shaughnessy, L. J. (1935). *J. Thorac. Surg.*, **5**, 386.

Pardee, H. F. B. (1920). *Arch. int. Med.*, **26**, 244.

Parkinson, J. and Bedford, D. E. (1927), *Heart*, **14**, 195.

Parry, C. H. (1799), Sycope Anginosa, Bath.

Robertson, W. B. (L959). *Lancet*, **1**, 444.

Towers, M. K. and Wood, P. (1958). *Brit. Med. J.*, **2**, 1067.

Vineberg, A. (1946), (1954). *J. Int. Coll. Surg.*, **22**, 503.

Yudkin, J. (1957). *Lancet*, **2**, 155.

Zoll, P. M., Wessler, S., and Blumgart, H. L. (1951). *Amer. J. Med.*, **11**, 331.

## Figures and Tables

**Fig. 1. f1:**
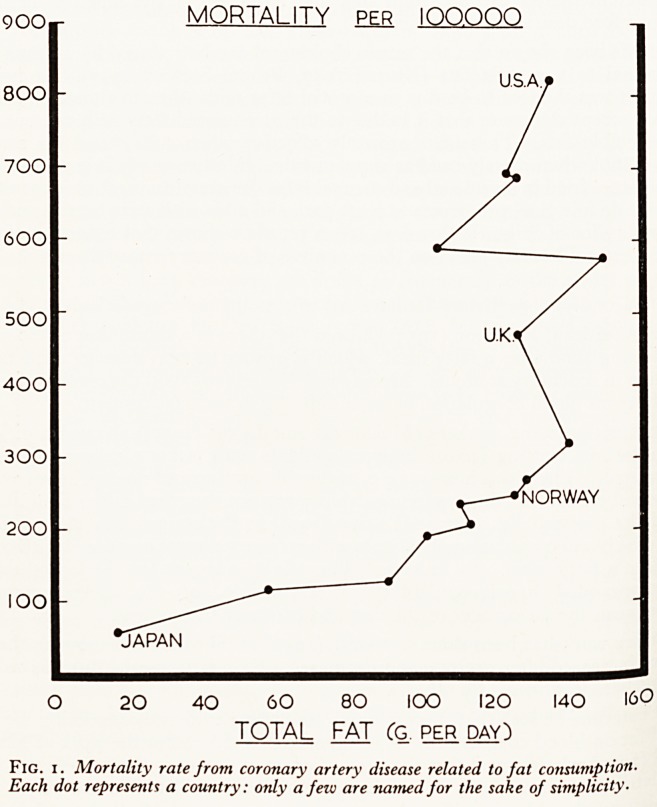


**Fig. 2. f2:**
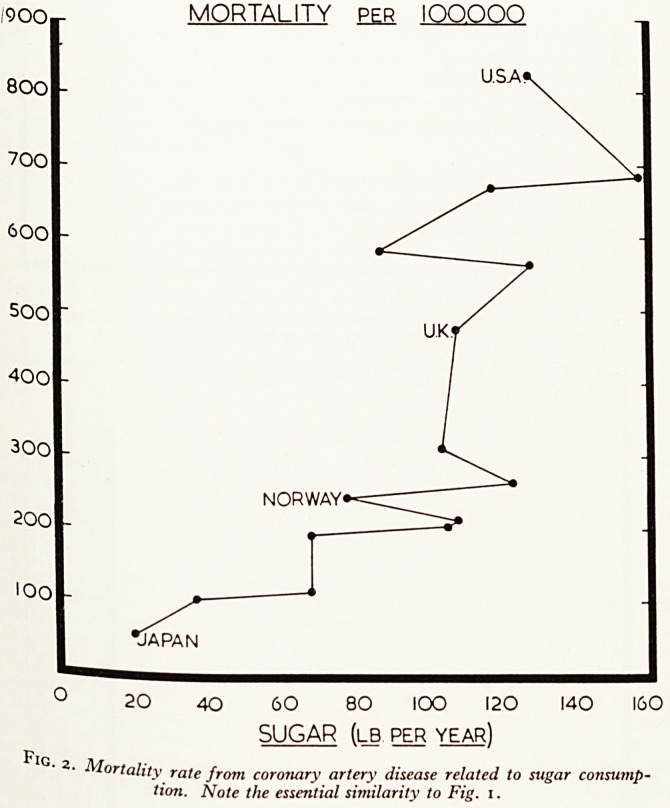


**Fig. 3. f3:**
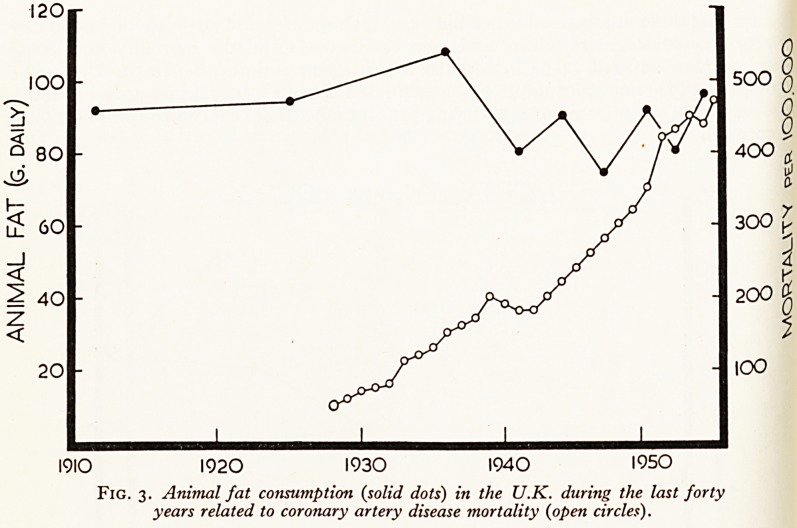


**Fig. 4. f4:**